# Design Optimization of Explosion-Resistant System Consisting of Steel Slab and CFRP Frame

**DOI:** 10.3390/ma14102589

**Published:** 2021-05-16

**Authors:** Jung J. Kim

**Affiliations:** Department of Civil Engineering, Kyungnam University, Changwon-si 51767, Korea; jungkim@kyungnam.ac.kr; Tel.: +82-55-249-6421; Fax: +82-505-999-2165

**Keywords:** optimization, reliability, explosion, explosion-resistant system

## Abstract

This study presents an explosion-resistant hybrid system containing a steel slab and a carbon fiber-reinforced polymer (CFRP) frame. CFRP, which is a high-strength material, acts as an impact reflection part. Steel slab, which is a high-ductility material, plays a role as an impact energy absorption part. Based on the elastoplastic behavior of steel, a numerical model is proposed to simulate the dynamic responses of the hybrid system under the air pressure from an explosion. Based on this, a case study is conducted to analyze and identify the optimal design of the proposed hybrid system, which is subjected to an impact load condition. The observations from the case study show the optimal thicknesses of 8.2 and 7 mm for a steel slab and a ϕ100 mm CFRP pipe for the hybrid system, respectively. In addition, the ability of the proposed hybrid system to resist an uncertain explosion is demonstrated in the case study based on the reliability methodology.

## 1. Introduction

Military vehicles are usually armored against explosions or impact loads. In the armor industry, steel appears to be the most commonly used material so far [1], besides others such as depleted uranium, which are also used to reinforce armor to carry low loads with high density. In addition, there is also a demand for lightweight materials for lighter, faster and safer military vehicles. Recently, an efficient material substitute for steel has been carbon fiber-reinforced polymer (CFRP), which contains numerous unique characteristics such as high strength-to-weight ratio and high strength [2], high energy absorption capacity, and corrosion resistance [3,4,5,6,7,8,9,10]. Moreover, the tensile strength and stiffness of steel structure were improved significantly by CFRP [11].

From another perspective, when an explosion takes place, an explosion-resistant object must receive a fairly large amount of energy in a very short time. An explosion wave is suggested to contain almost all the released energy from the explosion. For describing and characterizing the explosion wave over time, the values of overpressure above a specific impulse and surrounding environmental pressure are taken into account. The overpressure is reduced because the explosion wave travels further away from the explosion source [12,13,14]. For practical modeling, it is worth considering the material behavior under the strain loading rate [15,16,17,18]. Because deterministic explosion simulation cannot provide a powerful design, given the considerable sensitivity of explosion simulation to many unclear parameters controlling the explosion [19], a reliability-based approach for designing explosion-proof composites has been suggested [20,21,22,23]. The literature review showed the tremendous application of CFRP on strengthening old structures as well as independent members for resisting explosive impact. The use of CFRP for retrofitting reinforced concrete (RC) members such as slabs, beams, piers, and columns subjected to different explosion conditions was reported [24,25,26,27]. The observations showed the good effect of CFRP in improving the bearing capacity and energy absorption and reducing the deformation of RC members. In addition, the work of Wang et al. [28] presented an analysis of the deformation and ultimate strength of a CFRP–epoxy–steel hybrid beam under blast loading. The results showed the role of CFRP layers in increasing the dynamic load-bearing capacity and reducing the deformation due to the blast impact of the hybrid beam. In addition, the observations from the work of Mokhtaru and Nia [29] showed the proper protective design of steel pipelines by CFRP against the subsurface explosion. Above all, CFRP has demonstrated its role in improving the explosion resistance of hybrid structures.

In this present work, for observing the dynamic behavior of a hybrid structure subjected to atmosphere pressure generated by an uncertain explosion, a dynamic model is proposed. For an uncertain explosion, reliability analysis is taken to evaluate the failure probability of each member. By applying a high probability of failure to the steel slab and a relatively low probability of failure to the CFRP frame, the optimal thickness of the composite structure is observed. A case study for the design of a hybrid system containing a steel slab and a CFRP frame subjected to an uncertain explosion is conducted and observations are discussed. The observations from the present work showed the optimal design of an explosion-resistant hybrid system containing a steel slab and a CFRP frame. The ability of the proposed system to resist an uncertain explosion is demonstrated in the case study based on the reliability methodology.

It is noticeable that the structural behavior of CFRP was examined in this work theoretically. For practical application of this work, the stacking sequence and manufacturing details of the CFRP should be considered.

## 2. Methods

### 2.1. Dynamic Model for the Hybrid System Containing Steel Slab and CFRP Frame

**Figure 1:**
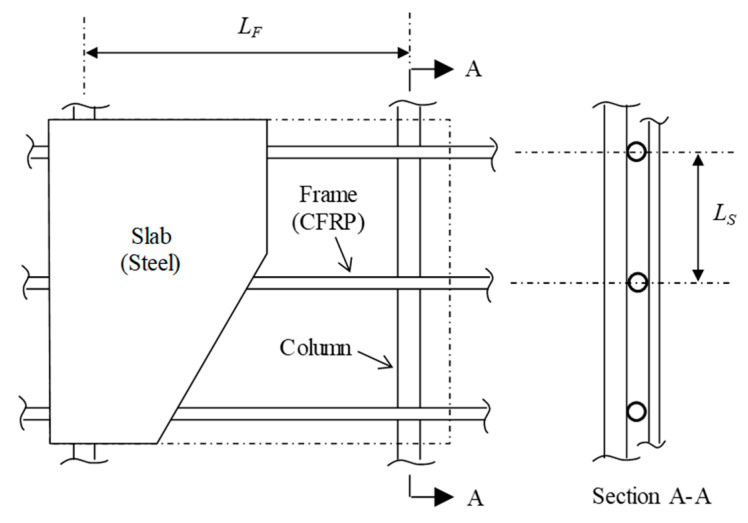
Steel slab and CFRP frame system.

A model was developed for analysis of the hybrid system containing a steel slab and a CFRP frame that was subjected to air pressures from an explosion, as shown in Figure 1.

**Figure 2:**
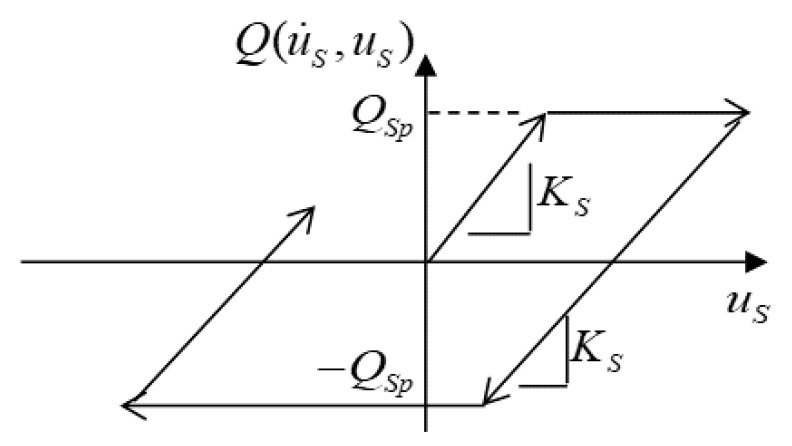
Idealized elastoplastic model of steel slab in a hybrid system.

For modeling of the hybrid system containing a steel slab and a CFRP frame, subjected to air pressures from an explosion, the elastoplastic dynamic behaviors of the system including mass, damper, and spring [30], are taken into account. The steel slab, which receives the explosion pressure, is attributed to the absorbing part [31] and the CFRP frame is attributed to the reflecting part. Therefore, an elastic model is applied for modeling the basic response of the CFRP frame, and an idealized elastoplastic model is applied for that of steel slab, as shown in Figure 2.

**Figure 3:**
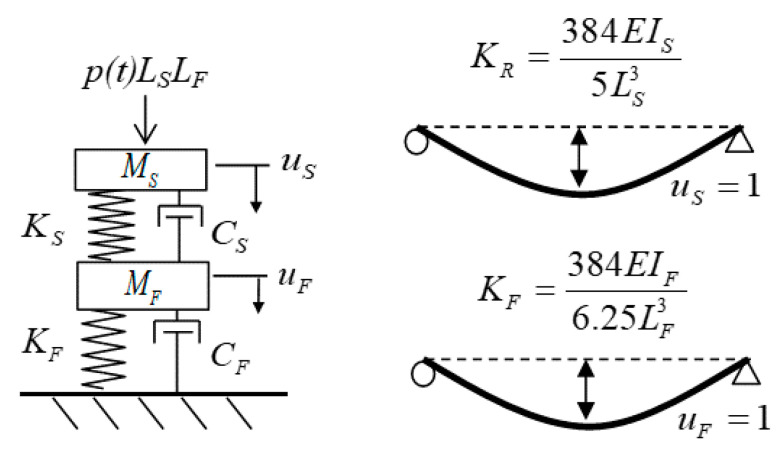
Explosion modeling of the steel slab and CFRP frame system.

Figure 3 presents a two lumped mass system, which is applied for modeling the hybrid system containing a steel slab and a CFRP frame. The motion equation of this system is expressed as:

(1) $$\begin{array}{l} \left[\begin{array}{cc} M_{S} & 0\\ 0 & M_{F} \end{array}\right]\left\{\begin{array}{c} {\ddot{u}}_{S}\\ {\ddot{u}}_{F} \end{array}\right\}+\left[\begin{array}{cc} C_{S} & -C_{S}\\ -C_{S} & C_{S}+C_{F} \end{array}\right]\left\{\begin{array}{c} {\dot{u}}_{S}\\ {\dot{u}}_{F} \end{array}\right\}\\ \;+\left[\begin{array}{cc} Q({\dot{u}}_{S},u_{S})/u_{S} & -Q({\dot{u}}_{S},u_{S})/u_{S}\\ -Q({\dot{u}}_{S},u_{S})/u_{S} & Q({\dot{u}}_{S},u_{S})/u_{S}+K_{F} \end{array}\right]\left\{\begin{array}{c} u_{S}\\ u_{F} \end{array}\right\}=\left\{\begin{array}{c} p(t)L_{S}L_{F}\\ 0 \end{array}\right\} \end{array}$$

where *u*, $\dot{u}$ and $\ddot{u}$ in that order are the displacement, velocity and acceleration of the steel slab and CFRP frame (see Figure 3). C and M mean the damping constant and the mass, respectively. $Q\left({\dot{u}}_{S},u_{s}\right)$ means the resisting force according to the applied strain rate regarding elastoplastic constitutive of steel, as shown in Figure 2. *K_F_* means the stiffness of the frame computed by considering the frame as the center support of the continuous two-span beam. *p*(*t*) means the applied explosion pressure to the steel slab, which considers the exposure time. Subscript “*S*” and “*F*” stand for the corresponding properties of the steel slab and the CFRP frame, respectively.

For our concern, Equations (2) and (3) below compute the maximum flexural strain *ε_s_*_,max_ in the steel slab and the maximum flexural stress *f_F_*_,max_ in the CFRP frame during the response time:

(2) $$\varepsilon_{S,\max}=\max\left\{\frac{24\left(u_{S}-u_{F}\right)t_{S}}{5L_{S}^{2}}\right\}$$

(3) $$f_{F,\max}=\max\left\{\frac{1.25(K_{F}u_{F})L_{F}d_{F}}{16I_{F}}\right\}$$

### 2.2. Reliability Analysis

For incorporating the uncertainties in applied explosion pressure into a system as well as the mechanical properties of the system’s materials into the design of the structure, reliability analysis has been conducted [32,33]. Critical states of the steel slab and CFRP frame are assigned to set up unexpected conditions or failures for the system.

The critical state of CFRP can be defined as:

(4) $$G_{F}=f_{\max}-f_{F,\max}$$

where *f*_max_ means the maximum elastic stress of CFRP and *f_F_*_,max_ means the maximum stress in the CFRP frame during the dynamic response to an explosion wave to reflect all the energy applied to the frame.

It is worth considering that the use of steel is for absorbing the applied explosion energy in the system as strain energy, and the yield state of the steel slab must be reached. If the strain energy capacity of steel is incapable of absorbing the strain energy from an explosion, the slab’s response is unpredictable. Therefore, the critical state *G_S_* of the steel slab is defined as:

(5) $$G_{S}=\varepsilon_{rupture}-\varepsilon_{S,\max}$$

where *ε_rupture_* means the failure strain of steel. *ε_rupture_* will take place when *G_S_* ≤ 0. The integration of joint probability density functions (PDF) of *u_Limit_* and *u_s_*_,max_ for the violation region of the critical state *G_S_* < 0 will give the probability of failure of the steel slab. Following that manner, a similar computation is conducted for the failure probability of the CFRP frame. In the present work, the reliability index *β* is taken into account instead of the failure probability for simplifying the design problem. The relationship between the reliability index and failure probability is described as:

(6) $$\beta =-\Gamma (p_{f})$$

where Γ means the inverse of the standard normal cumulative density function (CDF). 

### 2.3. Reliability Index and Design Optimization

The optimization process is established to determine the optimal dimension of the steel slab and CFRP frame system to observe a certain level of reliability of the hybrid system subjected to an uncertain explosion. The optimal thicknesses of the steel plate and the ϕ100 mm CFRP pipe give the expected reliability index, denoted as *β_S_* and *β_F_* for the steel slab and CFRP frame, respectively. The optimization problem can be posed as:

(7) $$\min\{{\left(\beta_{S,\mathrm{target}}-\beta_{S}\right)}^{2}+{\left(\beta_{F,\mathrm{target}}-\beta_{F}\right)}^{2}\}$$

In the present work, the thickness range of the steel plate for the slab was selected from 8 to 9 mm and that of the ϕ100 mm CFRP pipe for the frame was selected from 5 to 10 mm. 

## 3. Case Study

The mechanical properties of the steel and CFRP for the case study are presented in Table 1; *L_f_* = 2 m, *L_s_* = 1 m. 

The numerical simulations were performed using MATLAB^®^. To simulate the explosion pressure, the Friedlander decay function [12,20,34,35] is used here, such as

$$p(t)=p_{\max}\left(1-\frac{t}{t_{d}}\right)\exp\left(\frac{-\alpha t}{t_{d}}\right)$$

where *p_max_* means the maximum amplitude of the positive pressure caused by the explosion, *t_d_* means the time period of the positive pressure and *α* means the shape factor for the explosion model.

**Figure 4:**
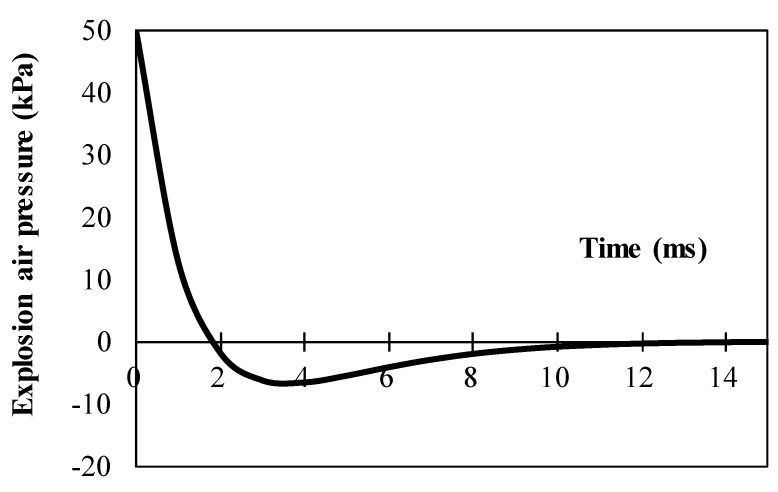
Friedlander explosion air pressure wave (*p_m_* = 50 kPa and *t_d_* = 1.8 ms).

The assumed explosion released an incident pressure of 50 kPa on the exposed surface of the hybrid system. The choice of the explosion incident was performed with the purpose to produce remarkably high stress in the resisting structure. In this model, *t_d_* and α are chosen as 0.0018 s and 1.0, respectively. Figure 4 presents the explosion model in this study.

The dynamic responses of the hybrid system caused by the explosion load were analyzed complying with the thicknesses of the steel plate and the ϕ100 mm CFRP pipe varying from 9 to 10 mm and from 5 to 10 mm, respectively. According to [36], the damping coefficients for both the steel and CFRP are assumed as viscous damping $C=2\zeta \sqrt{KM}$. Hereby, the damping ratio of 1% is used for both steel and CFRP. In addition, it is worth assuming that the connection between the steel slab and CFRP frame is held extremely well during the explosion loading.

**Figure 5:**
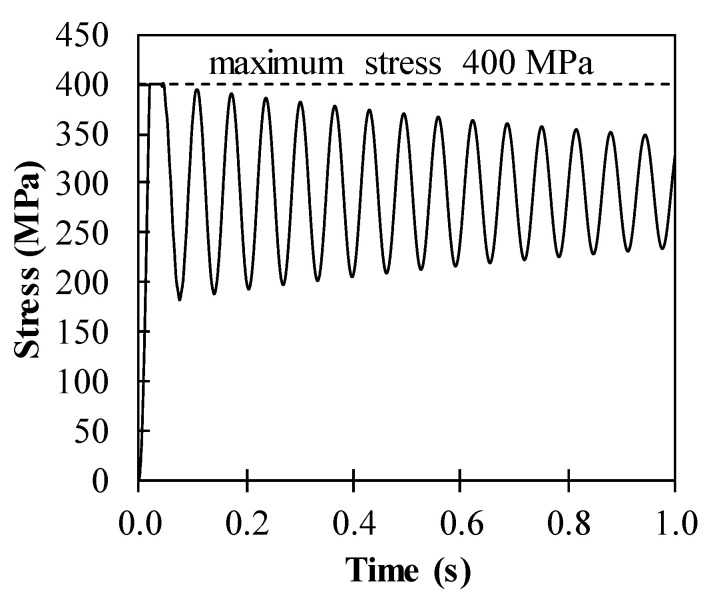
Stress development in steel of the hybrid system containing 8.4 mm thick steel slab and 8.0 mm thick ϕ100 mm CFRP frame.

The maximum flexural stress in the steel slab is limited in the yield strength of steel as 400 MPa. For example, the dynamic response of stress in steel is presented in Figure 5 for the hybrid system with the 8.4 mm thick steel slab and the 8.0 mm thick ϕ100 mm CFRP frame. As expected, the stress in steel is governed by the yield strength of 400 MPa. 

**Figure 6:**
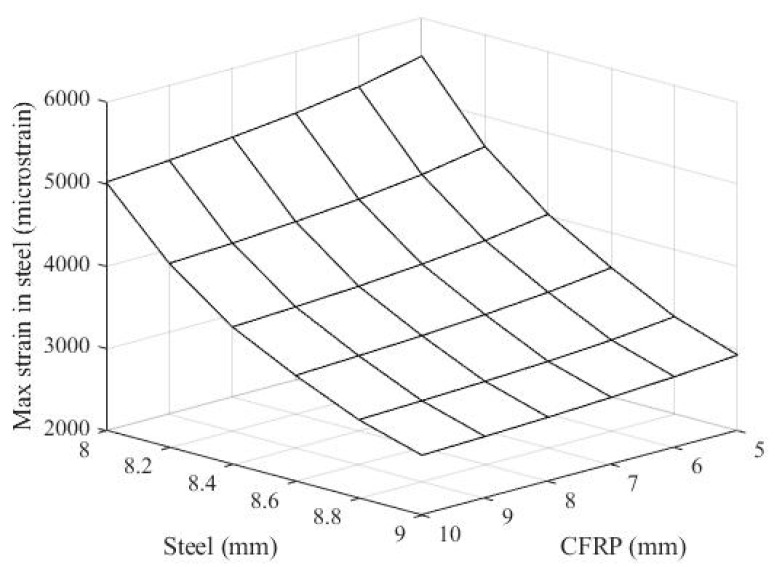
The maximum strain in steel with regard to the thicknesses of both steel slab and CFRP pipe.

**Figure 7:**
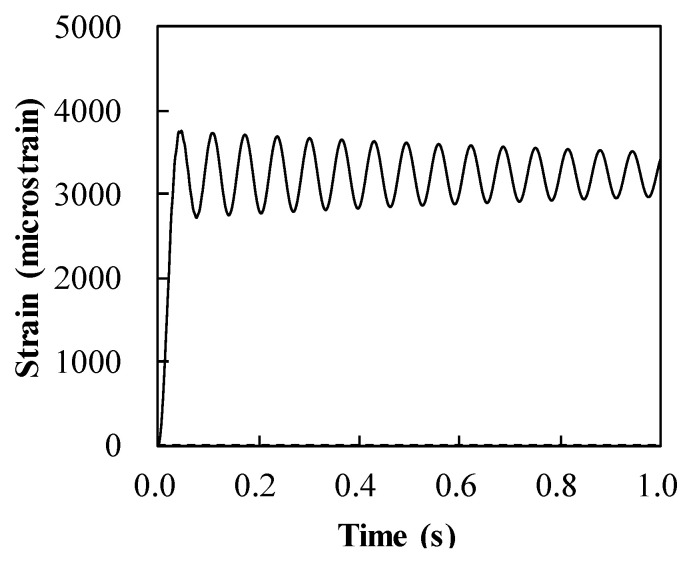
Strain development in steel of the hybrid system containing 8.4 mm thick steel slab and 8.0 mm thick ϕ100 mm CFRP frame.

Figure 6 shows the maximum strain in steel with regard to the thicknesses of both the steel slab and CFRP pipe. The dynamic response of strain in the steel of the resisting structure, consisting of the 8.4 mm thick steel slab and the 8.0 mm thick ϕ100 mm CFRP frame, is also presented in Figure 7. In Figure 6, the decrease in the maximum strain corresponded with the increase in thickness of the steel. However, this observation shows the insensitivity of the maximum strain in steel to the change in thickness of the ϕ100 mm CFRP pipe. The maximum strain of steel is computed under its rupture strain of 11% (11,000 microstrain) for all chosen analysis regions, as shown in Figure 6.

**Figure 8:**
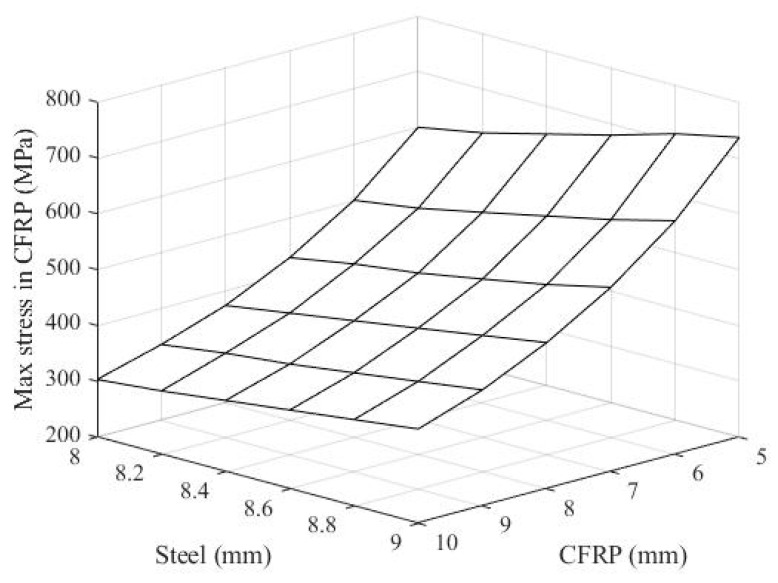
The maximum stress in CFRP with regard to the thicknesses of both steel slab and CFRP pipe.

**Figure 9:**
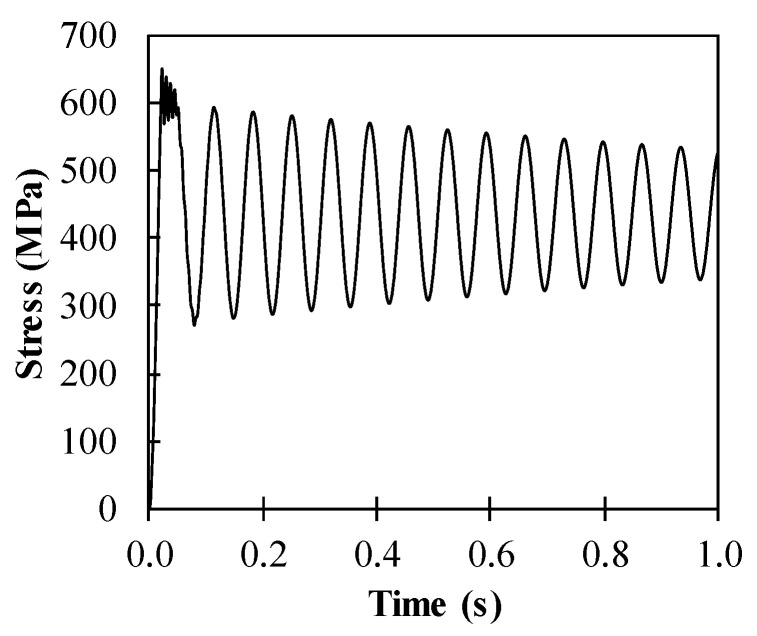
Stress evolution in CFRP of the hybrid system containing 8.4 mm thick steel slab and 5.0 mm thick ϕ100 mm CFRP frame.

Figure 8 shows the maximum stress in CFRP with regard to the thicknesses of both the steel slab and CFRP pipe. The dynamic stress in the CFRP of the resisting structure, consisting of the 8.4 mm thick steel slab and 5.0 mm thick ϕ100 mm CFRP frame, is also presented in Figure 9 as an example. The observation from Figure 8 shows a decrease in the maximum stress in CFRP when increasing the thickness of the ϕ100 mm CFRP frame and decreasing the thickness of the steel.

Considering uncertain explosions, the amplitude of explosion pressure p_m_, the rupture strength of the CFRP, and the rupture strain of the steel were assumed to be normally distributed with different coefficients of variation (COV) of 30% [37], 20%, and 25%, respectively.

**Figure 10:**
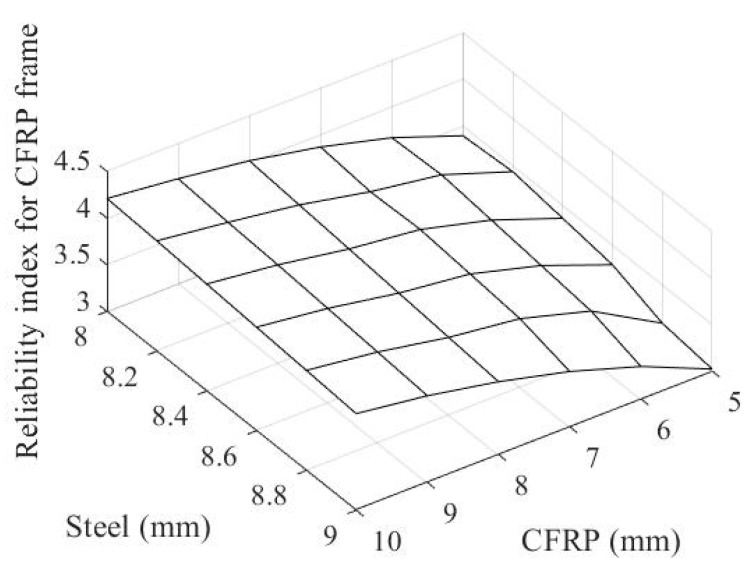
Reliability index of CFRP frame.

**Figure 11:**
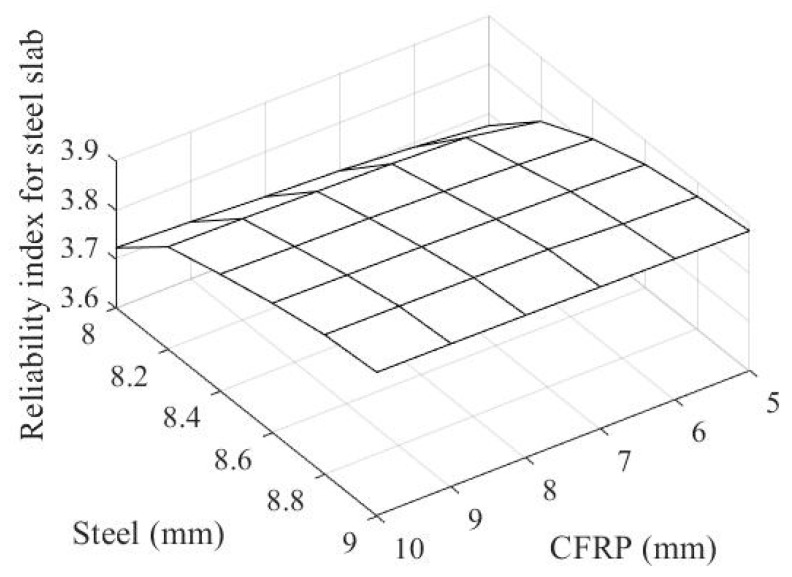
Reliability index of steel slab.

By assigning these uncertain coefficients to the threshold state functions in Equations (4) and (5), Figure 10 and Figure 11 show the reliability index for the failure probabilities of steel and CFRP with regard to thickness, respectively. For evaluating the failure probability, one thousand random variates for the magnitude of explosion pressure are generated. For each set of thicknesses of both the steel slab and CFRP pipe, the same set of magnitude of explosion pressure variables is taken into account to observe a consistent response surface. 

## 4. Results and Discussion

By applying these uncertainties to the limit state functions in Equations (4) and (5), the failure probability for the steel slab and CFRP frame with regard to the thicknesses of both the steel slab and ϕ100 mm CFRP pipe are computed and formulated as a reliability index in Figure 10 and Figure 11, respectively. 

The reliability index for both CFRP frames, as shown in Figure 10, increases with the increase in the ϕ100 mm CFRP pipe’s thickness, while it decreases with the increase in the steel slab’s thickness. In Figure 11, the reliability index for the steel slab increases with the increases in the thicknesses of both the steel slab and ϕ100 mm CFRP pipe. To observe the optimal thicknesses of the steel slab and ϕ100 mm CFRP pipe for an uncertain explosion using Equation (7), the following design reliability index for both materials was considered: *β*_target_ = 3.8 (corresponding to a probability of failure of 0.0072%). The reliability index for conventional strength design was applied to the steel slab and CFRP frame.

**Figure 12:**
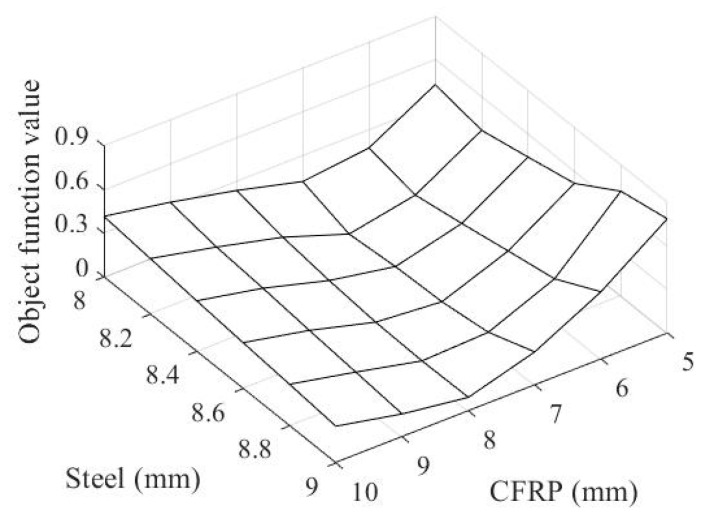
Objective function surface.

**Figure 13:**
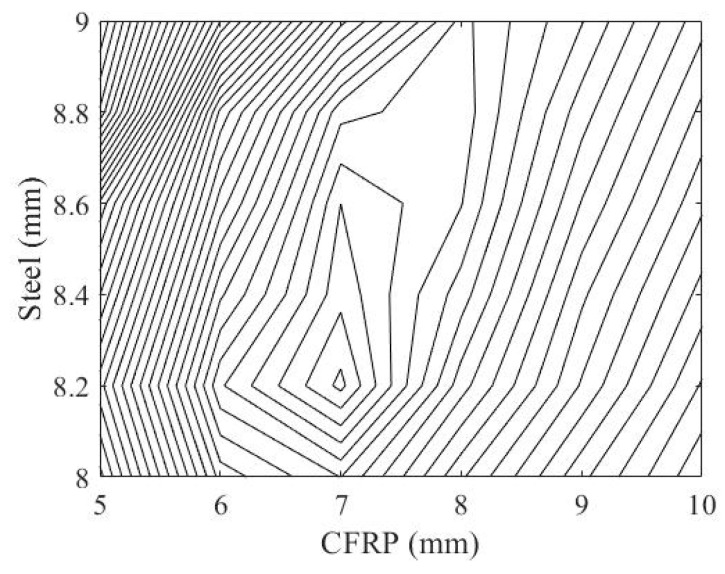
Contour plot of the objective surface in Figure 12.

The result in Figure 12 is obtained by computing the objective function in Equation (7). Based on this result, the optimal set of 7 mm thick ϕ100 mm CFRP pipe and 8.2 mm thick steel slab for the hybrid system can be determined. Figure 13 presents the corresponding contour plot obtained from this result.

If there more than two optimal solutions exist due to the significant non-linearity of the beta surfaces, the optimal solution can be obtained by reducing the use of materials.

**Figure 14:**
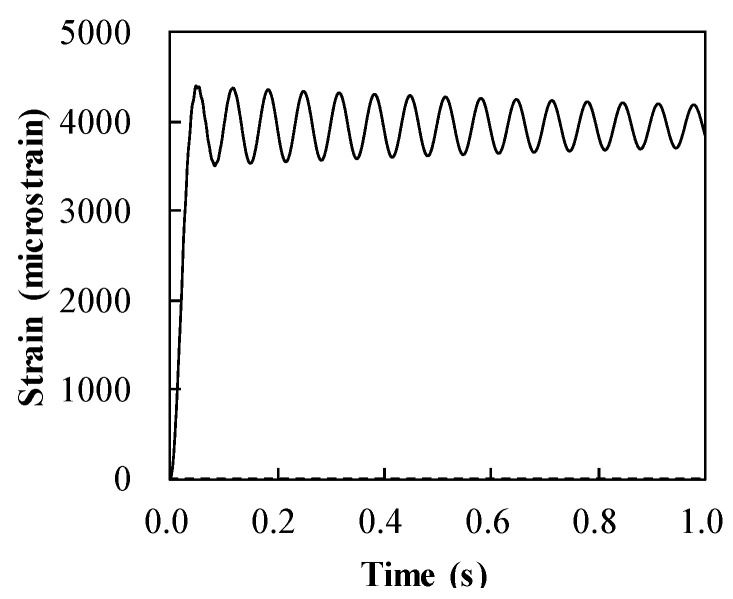
Strain evolution in steel of the optimized hybrid system containing 8.2 mm thick steel slab and 7.0 mm thick ϕ100 mm CFRP frame.

**Figure 15:**
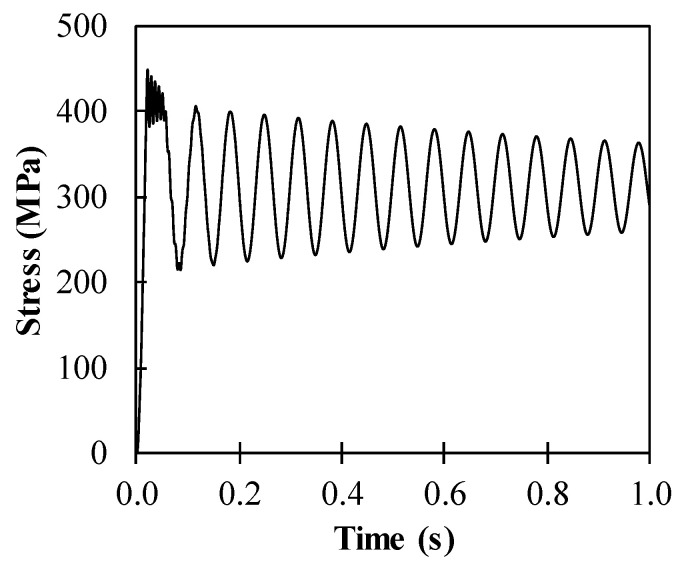
Stress evolution in CFRP of the optimized hybrid system containing 8.2 mm thick steel slab and 7.0 mm thick ϕ100 mm CFRP frame.

The optimal hybrid system to resist uncertain explosion, with a mean maximum pressure of 50 kPa and 30% COV, is then pointed out to have an 8.2 mm thick steel slab with a 7.0 mm thick ϕ100 mm CFRP pipe. The dynamic responses, steel slab strain and CFRP frame stress evolutions, for the optimized composite structure, are shown in Figure 14 and Figure 15, respectively. The remaining strain of steel reaches 0.44%, while CFRP frame stress approaches 448 MPa. Previously, the optimal hybrid system containing titanium and aluminum that was subjected to an explosion source was considered [21]. The titanium layer and the aluminum layer were simulated as reflecting and absorbing members, respectively. Moreover, the optimal hybrid system containing CFRP and steel layers that was subjected to the same explosion source was also found with the present work [23]; the CFRP layer was considered as a reflecting layer, and the steel layer was considered as an absorbing layer. The observation from the previous work showed optimal thicknesses of 8 and 138 mm for the CFRP and steel layers, respectively. As a comparison, the hybrid system proposed in this present work showed higher effectiveness in reducing the use of materials. Therefore, reducing the mass of the structure leads to a flexible application for armored military vehicles or structures.

It is worth noting that a realistic explosion will not generate uniform pressure on the exposed surface of the composite slab, and the materials’ yield typically happens locally. Therefore, conducting finite element (FE) analysis to examine the effect of the explosion on the composite structure in order to observe the detailed dynamic behaviors is necessary. Following that manner, large deformation conditions, continuous models, and interface conditions should be used in order to consider detailed dynamic behavior. The above method provides a simplified approach for observing the initial optimal design of the hybrid resisting structure.

## 5. Conclusions 

In this study, the design optimization of an explosion-resistant hybrid system based on the simplified reliability design method is presented in conjunction with a case study. The hybrid system contained a steel slab and a CFRP frame to absorb and reflect the explosion energy. The same target reliability index level is considered for both materials. The specific design of a steel slab–CFRP frame hybrid system subjected to an uncertain explosion is presented. The optimized design hybrid system proved its ability to resist the explosion. As strain rate-dependent material properties were neglected, the proposed design method might be considered as a conservative design methodology for a hybrid system to improve explosion resistance.

## Figures and Tables

**Table 1:** Mechanical properties of CFRP and steel for the proposed composite structure.

Material	Properties			
	Density (kg/m^3^)	Strength (MPa)	Modulus (GPa)	Rupture Strain
CFRP	1800	2000	245	
Steel	7800	400	210	11%

## Data Availability

The data presented in this study are available on request from the corresponding author.
